# Model of the Vibration Signal of the Vibrating Sieving Screen Suspension for Condition Monitoring Purposes

**DOI:** 10.3390/s21010213

**Published:** 2020-12-31

**Authors:** Anna Michalak, Jacek Wodecki, Michał Drozda, Agnieszka Wyłomańska, Radosław Zimroz

**Affiliations:** 1 Faculty of Geoengineering, Mining and Geology, Wroclaw University of Science and Technology, Na Grobli 15, 50-421 Wroclaw, Poland; jacek.wodecki@pwr.edu.pl (J.W.); radoslaw.zimroz@pwr.edu.pl (R.Z.); 2 KGHM Polska Miedz SA, Oddzial Zaklady Wzbogacania Rud, Kopalniana 1, 59-101 Polkowice, Poland; Michal.Drozda@kghm.com; 3 Faculty of Pure and Applied Mathematics, Hugo Steinhaus Center, Wroclaw University of Science and Technology, Wybrzeże Wyspiańskiego 27, 50-370 Wroclaw, Poland; agnieszka.wylomanska@pwr.edu.pl

**Keywords:** vibrating sieving screen, vibration, modelling

## Abstract

Diagnostics of industrial machinery is a topic related to the need for damage detection, but it also allows to understand the process itself. Proper knowledge about the operational process of the machine, as well as identification of the underlying components, is critical for its diagnostics. In this paper, we present a model of the signal, which describes vibrations of the sieving screen. This particular type is used in the mining industry for the classification of ore pieces in the material stream by size. The model describes the real vibration signal measured on the spring set being the suspension of this machine. This way, it is expected to help in better understanding how the overall motion of the machine can impact the efforts of diagnostics. The analysis of real vibration signals measured on the screen allowed to identify and parameterize the key signal components, which carry valuable information for the following stages of diagnostic process of that machine. In the proposed model we take into consideration deterministic components related to shaft rotation, stochastic Gaussian component related to external noise, stochastic α-stable component as a model of excitations caused by falling rocks pieces, and identified machine response to unitary excitations.

## 1. Introduction

**Figure 1:**
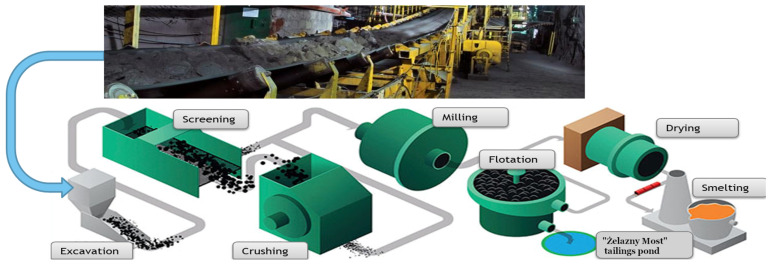
The processing scheme.

Demand for raw materials is growing due to the rapid development of advanced technologies. Rare Earth materials are the most critical, however, well-known copper becomes again a crucial material due to, for example, e-mobility development. Production of copper is a complex process, nowadays raw materials mining is still a primary way to acquire copper ore, it requires further processing. The extraction of copper ore in Poland is focused around three underground mines owned by KGHM, which are located in Lubin, Rudna, and Polkowice–Sieroszowice. The averaged copper content in the ore is c.a. 2% so it has to be enriched to obtain 15–20% of copper in the processed ore. This process is complicated and covers, among others: crushing, sieving, milling, which are examples of so-called mechanical processing of raw materials. It is done in the concentrator plants, that are located near each mine and cooperate with them. The description of further chemical processing of the ore is intentionally skipped here (see Figure 1). Mechanical processing of raw materials requires reliable machines as a sieving screen for material classification by size of particles, crushers for oversized pieces fragmentation, and mills for providing fine particles—appropriate for chemical processing, i.e., flotation. Due to specific processes, maintenance of all these machines is challenging.

### 1.1. Machines for Raw Materials Processing

All three components in this specific production line have the same challenging issue. Due to the process of screening, crushing, or milling, the processing of raw materials provides a lot of random shocks. It is related to the striking of materials against the walls of the machine or ore fragmentation process. Crushers have been discussed in our earlier works [1,2]

The machine investigated here is a vibrating sieving screen. As mentioned before—a sieving screen is the first critical element of the raw materials processing chain. After the ore is delivered to the plant, it is directed to the screen for classification based on the size of ore pieces.

The ore is divided into two products: pieces of small size (<40 mm) and large size (>40 mm) [3]. Fine-grained product is fed to ball mills for grinding. Coarse product has too large pieces for milling, and could potentially damage the mills from the inside, since they are not designed to deal with larger lumps. Hence, it is directed to hammer crushers, where it can be further fragmented below 30 mm. Then it can be safely transferred to milling. In the next stages, flotation allows extracting copper particles from the fine ore dust. In this way the concentrate is obtained, that after drying is transferred to smelting plants [4]. Process flow is visualized in Figure 1.

### 1.2. Processing, Modeling, and Analysis of Vibrations—A Brief State of the Art

A condition monitoring often requires advanced signal processing techniques for: pre-processing, features extraction, detection, etc. There are several interesting review papers in the field of condition monitoring and fault diagnostics for industrial processes in general [5]. One can also find several comprehensive reviews of model-based condition monitoring regarding the popular objects such as wind turbines [6,7] or bearings [8]. There are many works that exploit so-called machine learning [9], artificial intelligence [10], or deep learning-based approaches [11] to data classification and recognition of machinery condition. Another perspective is related to knowledge about phenomena.

Model-based diagnostics (MBD) is a widely used approach in the field of maintenance management of industrial machinery, where it is important to distinguish dynamics-related models [3,12,13] as well as signal models [14,15,16,17,18,19]. Krot et al. described diagnostics of springs from the point of view of a dynamic model [3], and in this paper authors attempt an alternative approach using data-driven signal model. Naturally, it is crucial is to be able to use an appropriate model, that very often has to be prepared from scratch. The models used in MBD can be of various types, from logic-based models to differential equations [20,21]. Depending on the model type, different approaches to MBD can be used, for example discrete event systems approach [22], statistical approach [23], AI-based approaches [24], and approaches within the framework of control theory. Of course modeling is a widely used approach in various areas of research beside MBD, such as analytics in environmental sciences [25,26], economy [27,28], medicine [29,30] and many others. However, speaking specifically about creating signal models, one of the largest parts of the cognitive process involves decomposition of the real-life data for the individual component identification.

In this paper, the model we mean is the one of the vibration signal acquired from a sieving screen. Modeling of signals is commonly used in vibration analysis. In [31] Zhuge et al. proposed non-stationary modeling of vibration signals based on the time-variant of autoregressive (AR) model. If the modeled system is time-varying, the AR model appropriate for stationary signals can be extended to Time-Varying AR models. In [32] Poulimenos and Fassois proposed methods based upon time-dependent autoregressive moving average (TARMA) representations. They provided a critical survey and comparison of parametric time-domain methods for non-stationary random vibration modeling and analysis. Wang et al. [33] discussed various signal processing techniques (mostly related to the time-frequency domain) allowing the description of the signal as a set of coefficients or subsignals (as EMD components). In [34] Avendaño-Valencia and Fassois described the methods of modeling the stationary and non-stationary random vibration and their analysis for an operating wind turbine. It considers three stationary modeling methods: autoregressive (AR), and autoregressive moving average (ARMA) and Welch spectral estimation and five approaches for non-stationary cases: parametric modeling by means of smoothness priors (SPs), time-dependent autoregressive modeling, functional series (FS) time-dependent autoregressive modeling, adaptable functional series (AFS) time-dependent autoregressive modeling and non-parametric Wigner–Ville spectral correlation In [35] Jiang and Zhang propose to use the genetic algorithm to find the optimal parameters of vibration model and further applied them to the model-based diagnostic approach.

**Figure 2:**
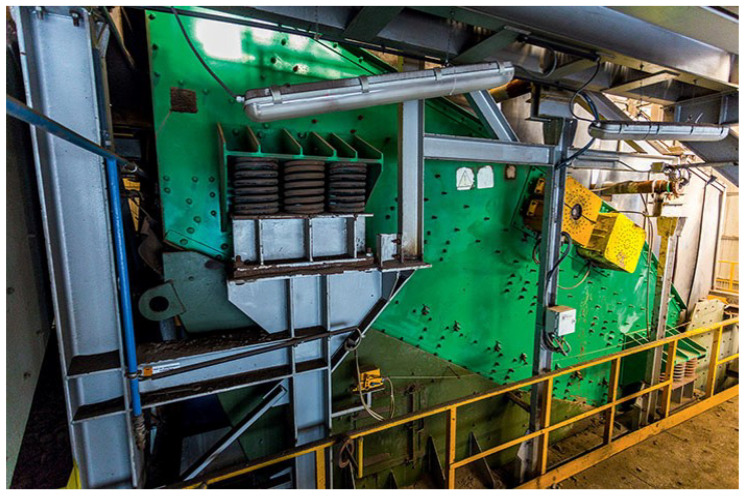
The vibrating screen.

In this paper, the authors focus on modeling the vibration signal measured on the sieving screen (see Figure 2). This machine is crucial for the ore enrichment process, it is the first stage of the process flow in the mineral processing plant. Its reliability is of utmost importance because if it fails, it can cause damage in subsequent devices, especially mills. Hence, there is a high demand from the industry for high-level monitoring and diagnostics of the screens. There are several elements of the screen that are especially prone to fail due to operational fatigue. The most important are the springs that suspend the machine [36,37], and bearings that hold the rotating shafts [38,39]. Due to the importance of this type of machine, attempts are made to optimize them at the stage of design based on previous experience [40,41]. In [42] Safranyik et al. proposed the optimal oscillation parameters of the vibration screens.

One of the most important features of a screen, especially from the point of view of the vibration-based approach is the fact that ore stream at the input of the machine contains some amount of oversized pieces, which upon hitting the machine decks generate strong impulsive excitation, that is manifested prominently in the vibration signal as short-time wideband impulses. Modeling of such behavior is a non-obvious task, that has to be solved using the formal mathematical description. To address this, the authors decide to model the occurrence of those large pieces using a random process with the α-stable distribution. The α-stable distribution belongs to the family of so-called heavy-tailed distributions, which means that it has a significant probability of generating outliers. Those outliers in the context of vibration data are useful for simulating random high-energy impulses. The α-stable distribution is an extension of the Gaussian one, in particular α-stable distribution becomes Gaussian for α=2. It is the four-parameter distribution, where the α parameter corresponds to the probability of the large observation (impulses) occurrence [43]. In the presented case, the α-stable random variable is related to the excitations caused by falling rocks pieces.

In this paper, we present the first attempt to construct the model of vibration signal of the industrial vibrating screen, in particular signal measured on the suspension of such machine. The main reason is the possibility of performing MBD of spring sets that carry the machine. Those springs in everyday operation are subjected to high-energy oscillatory vibrations that cause them to develop microcracks being an early sign of suspension failure. It is expected that MBD of such suspension can make it possible to detect a fault in the early stage of development. Due to the high cost of maintenance, the effort to provide early damage detection can allow to better schedule the repair tasks and result in substantial savings in comparison to the periodic preventive installation of new parts.

The proposed model can be used for model based approach, i.e., to compare real data with model identified using historical data. In fact the main motivation of this paper is to use model for various numerical experiments when testing different diagnostic methods. It was already mentioned, that unique machine, with critical importance in technological process, cannot be an object used for diagnostic experiments, we cannot introduce any damages there, we are not allowed to use various granulation of material (ore) stream etc. However, we can do as much we want when we have theoretical, tuned to reality, model of the signal. In our previous research [44] we have analysed effectiveness of cyclostationary analysis for copper ore crusher bearings. It is also good example showing that for some specific parameters of raw signal, the methods—well-founded theoretically—failed in practice. Using simulations we explained why it happens and we defined some limitations for using these methods. Without the model, this could be impossible.

## 2. Measurement Description

**Figure 3:**
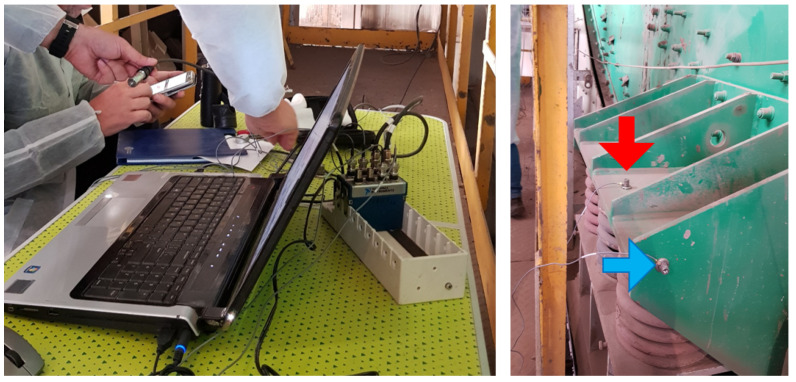
Computer and data acquisition card used for the experiment (**left image**) and positioning of horizontal and vertical direction sensors on the bottom right spring set (**right image**).

**Figure 4:**
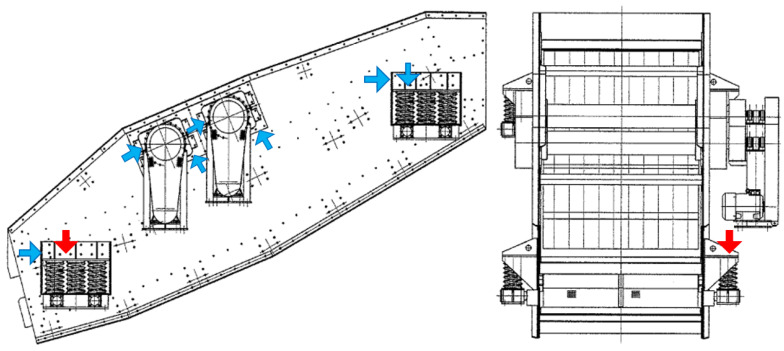
The positions of the vibration sensors used in the measurement session on the vibration screen. The left image presents machine’s left side, however the same sensors with identical configuration in the same positions were installed also on the right side, for the total amount of 16 sensors. The red arrow indicates the position of sensor that registered the signal used for this work, blue arrows indicate the positions of the rest of the sensors. In the right picture (front view) only the one sensor used in this work has been marked for image clarity.

The vibration signal used to construct the presented model has been acquired on the real-life sieving screen used in the mining industry for copper ore classification. The measurement has been performed using the National Instruments 9233 acquisition card and Endevco 751-10 accelerometers (see Figure 3). In total authors used 16 sensors mounted on the machine on eight components: four bearings and four sets of springs. On each components there is installed a pair of sensors, one in horizontal and one in vertical direction. The sensor of interest has been installed vertically, directly on one of the four spring sets that constitute the suspension of the machine. In particular, it was bottom right set looking from the front of the machine (see the red arrows in Figure 3 and Figure 4). Authors were interested in the motion of the machine from the point of view of diagnosing the suspension springs, so vertical vibration data of the springs (the specific direction of motion of the component of interest) is the exact measurement that authors needed. The sampling frequency of the measurement has been set to 25 kHz.

The considered screen of type SWR-3 PZ2-2.2-6.0 can accept ore pieces of dimensions up to 50×50×30 cm, which is derived from the dimensions of the grids at ore dumping points. The machine itself is almost 10 m long and weighs over 15 tons. Depending on the configuration, those types of screens can allow to process from 10 to over 1000 tons of material every hour.

## 3. Methodology

**Figure 5:**
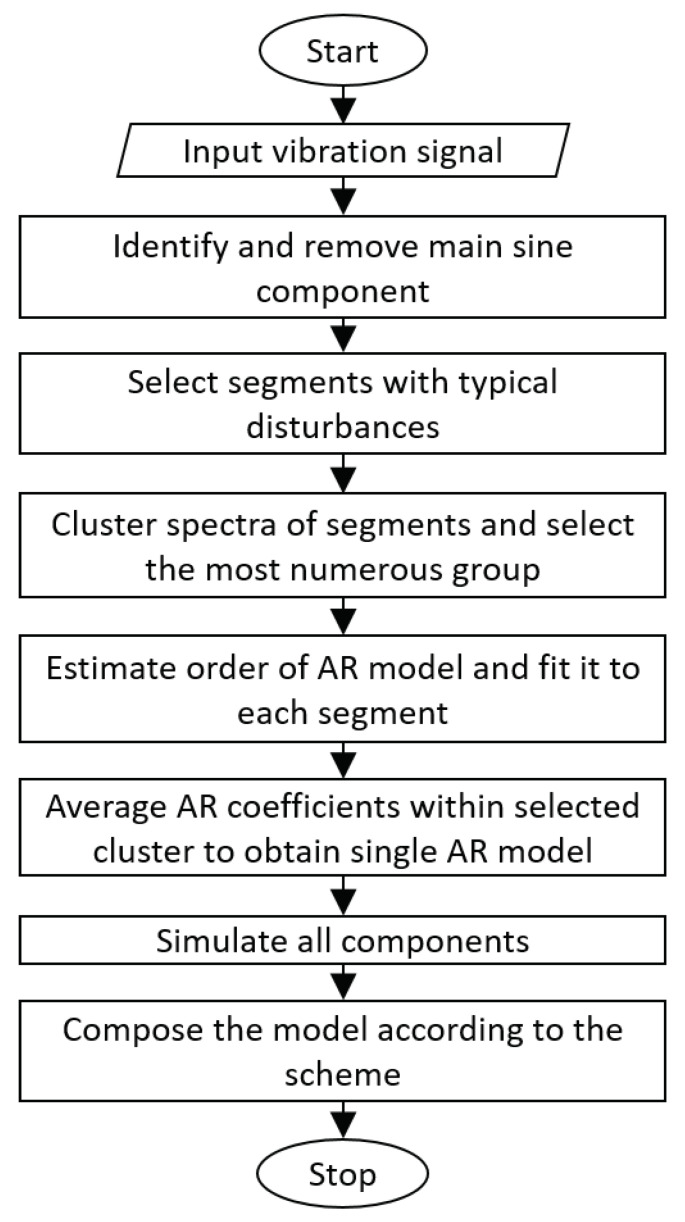
The flowchart of proposed model identification procedure.

In this section, we present each step of the proposed model identification procedure. The general scheme of the procedure is presented in Figure 5. Firstly, the measured vibration signal is loaded. Next, the highest energy deterministic component (related to the rotation of main shafts, which in practice manifests itself in the data as a singular sine wave) is identified using simple spectral analysis of the raw signal. If the deterministic component is present in the signal, it will appear as a discrete component in the spectrum. Then, frequency and amplitude are approximated from spectral representation of the signal and it is subtracted from the input signal as it is described in Section 3.1. After removing the deterministic component, it is assumed that the following features should be expected in the signal: (a) some random noise and (b) set of impulses related to shocks related to falling elements into the sieving deck. Next, the representative examples of disturbances are selected. Representative examples mean segments of signal with a single impulse in the segment. For each of these selected segments the autoregressive (AR) model is estimated. In the next step, vectors of coefficients are processed to obtain a representative model of disturbance. Details of this operation are described in Section 3.3. Finally, the individual components are simulated and the model is composed according to the scheme (see Figure 7).

### 3.1. Identification and Removal of the Main Sine Component

In the signal analysis, one of the first steps should be the identification and removal of the deterministic component, because it is strongly dominating the entire signal structure. In this case by “deterministic component” we understand the entire collection of elementary components that are deterministic in their nature and may be present in the signal. In the analyzed case, it is the collection of sine components that are related to the rotation of the shaft. Based on the spectrum of the input signal, we can identify the amplitude, the frequency, and the phase of those components. It allows us to describe and remove the sine components from the raw signal (for the deterministic components removal see also [45]. In this particular case, a single sine wave was overwhelming the structure of the signal, so for the simplicity of this model, we assumed that this is the component of interest.

In practice, this is a three-step operation:

- Calculating the Fourier spectrum of the signal [46],
- Finding the amplitude and frequency of the strongest component on the real (amplitude) part of the spectrum, and the phase value at the identified frequency on the imaginary (phase) part,
- Generating the identified component and subtracting it from the signal.

### 3.2. The Segments Selection

Next, from the de-trended signal, a transfer function of the machine has to be estimated. For the purpose of this operation, we assume that the impact of a piece of ore falling into the machine is a Dirac-like unitary excitation d(t), and the impulse X(t) registered by the sensor is a machine responding to the excitation via its transfer function H(t) according to the model:

(1) X(t)=d(t)*H(t).

**Figure 6:**
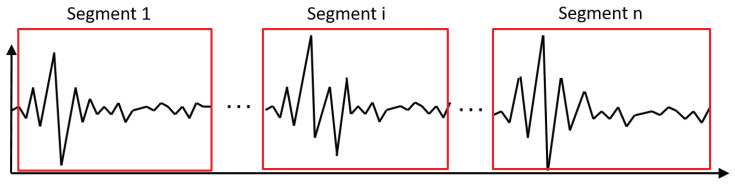
The idea of segment selection.

Note that the “*” operator means convolution of d(t) and H(t). Hence, we select *n* segments with representative examples of the impulses X(t) as in the Figure 6. The presented methodology is based on the modeling of the impulses, so it is expected that the impulse response of the transfer function will also be decaying.

For the time being, we decided to perform segmentation manually to be able to select only those impulses that are visible in the clearest and representative way. Future work assumes the development of this methodology to be able to perform the segmentation automatically [1,47,48].

### 3.3. The Estimation of the Autoregressive Model Coefficients

To obtain the response of the machine, we use the autoregressive (AR) model with order *p*. The AR(p) model is defined as follows [49]:

(2) $$X\left(t\right)=c+\sum_{i=1}^{p}\varphi_{i}X\left(t-i\right)+\varepsilon \left(t\right)$$

where $\varphi_{1},\cdots ,\varphi_{p}$ are the model coefficients, *c* is the constant and {ε(t)} is the white Gaussian noise with the variance $\sigma^{2}$ i.e., this is the sequence of uncorrelated Gaussian random variables with 0 mean and variance $\sigma^{2}$.

From the technical point of view, the AR model is designed as a multiband bandpass IIR (infinite impulse response) filter of very specific magnitude response, that approximates the frequency-response function of the data [50]. The $\varphi_{1},\cdots ,\varphi_{p}$ coefficients are estimated for each segment by using Yule-Walker method [49]. Because of the formula of the AR(p) model, the vector of parameters has the form $C=[1,-\varphi_{1},\cdots ,-\varphi_{p}]$. After this step of the procedure, we obtain the matrix containing *n* vectors (number of segments) of (p+1) coefficients (parameters $\hat{C}$ of the AR model).

In the next step, hierarchical cluster analysis is used for the spectra of the extracted segments [51]. The authors use clustering to obtain a class of segments with similar properties and use them to get the representative impulse response of the machine. In this case, a hierarchical method has been used, which seeks to build a hierarchy of clusters. There are two strategies that can be used in such cases [52]:

- **Agglomerative:** also known as “bottom-up” approach. In this scenario, every observation begins as its own cluster. As the algorithm progresses pairs of clusters closest to each other are merged into larger clusters.
- **Divisive:** also known as “top-down” approach. For this scenario, all observations begin as one cluster. As the algorithm progresses clusters are split recursively producing a larger amount of smaller clusters.

In this example, the authors used the agglomerative approach, since it is deterministic in its behavior, and the divisive approach very often needs to take advantage of other heuristics (i.e., k-means) to properly define splits. The agglomerative process is tracked, and as a result, it is possible to draw a dendrogram that illustrates all the connections between clusters and the way that they were merged. Based on that, one can select the level of precision of the clustering (in practice, when the merging stops, so the desired amount of clusters can be obtained). In this application Euclidean metric for distance and Ward linkage criterion were used [51].

To obtain the order *p* for the AR model, the authors performed the empirical quality test of the final result. It is based on calculating an error between the spectra of real and modeled signal. In the further analysis we denote the spectra as $A=\{a_{1},\cdots ,a_{m}\}$, $B=\{b_{1},\cdots ,b_{m}\}$, respectively. For this purpose the ordinary root mean squared error (RMSE) is used, which for compared vectors *A* and *B* of length *m* is defined as:

(3) $$RMSE\left(A,B\right)=\sqrt{\sum_{i=1}^{m}\frac{{\left(a_{i}-b_{i}\right)}^{2}}{m}}.$$

Spectrum A was prepared as a mean of spectra calculated from the segmented impulses belonging to the main class. Spectrum B was calculated from the signal obtained using an impulse response of a given AR model.

RMSE value was calculated for each comparison when AR order *p* takes a value between 30 and 400 with a resolution of 5. The authors decided not to start from p=1, because the shortest AR models would not be usable anyway.

A set of obtained RMSE values forms a quasi-convergent vector. The final AR order is selected as a point where the value of the error is smallest, such as:

(4) p=argmin(RMSE)

It is important to note that dedicated methods to establish the AR order are known (AIC, BIC, etc.). However, the authors decided to use the method described above [49,53]. Unfortunately, the best answer that was obtained was p=37, which turned out to be way too low to describe the real signal well enough. It was especially visible on the spectrograms where it was clear that important frequency components are missing. This shows that those methods are not as proper as one might think. This is also the reason that the authors set the starting value for order evaluation equal to 30, it is slightly smaller than 37, but the errors originating from spectra generated from AR models of orders *p* between 30 and 40 can be also included in the investigation.

**Figure 7:**
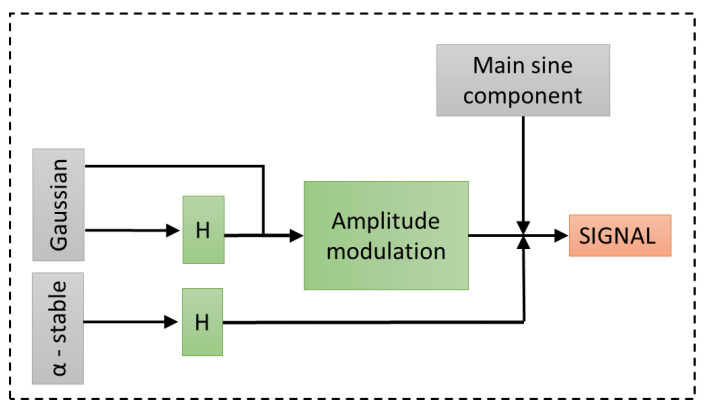
The scheme of proposed model construction.

Following the scheme presented in Figure 7, the only one vector of coefficients which allows to obtain the impulse response of the machine is needed. Hence, for each class, we average the vector of the obtained coefficient to obtain *k* vectors (corresponding to *k* classes) with (p+1) elements (corresponding to the number of estimated coefficients). Then, we select the most numerous class as a representative group, and we take it under consideration to obtain one vector corresponding to the response of the machine. The amount of classes *k* has been determined by the Silhouette criterion [54].

Calculated parameters of the AR model are used to construct the transfer function of the machines signal path. The transfer function H is represented in the following form:

(5) $$H\left(t\right)=\frac{1}{1-{\bar{\varphi }}_{1}t^{-1}-\cdots -{\bar{\varphi }}_{p}t^{-p}},$$

where $\left[1,-{\bar{\varphi }}_{1},\cdots ,-{\bar{\varphi }}_{p}\right]$ is the averaged vector of coefficients corresponding to the most numerous class.

### 3.4. The Signal Construction

To simulate the process of rocks falling into the machine, the authors used the α-stable distribution, which is characterized by four-parameters and denoted as S(α,β,σ,μ). It belongs to the class of continuous probability distributions. In literature, one can find a few equivalent definitions of the α-stable distribution [43,55,56]. The random variable *X* is called α-stable if its characteristic function is defined as follows:

(6) $$\begin{array}{c} E\left[e^{itX}\right]=\left\{\begin{array}{cc} \exp\left\{-\sigma^{\alpha }{\left|t\right|}^{\alpha }\left\{1-i\beta \mathrm{sign}\left(t\right)\tan\left(\pi \alpha /2\right)\right\}+i\mu t\right\} & for\;\alpha \neq 1,\\ \exp\left\{-\sigma |t|\{1+i\beta \mathrm{sign}\left(t\right)\frac{2}{\pi }\log(|t|)\}+i\mu t\right\} & for\;\alpha =1, \end{array}\right. \end{array}$$

where α∈(0,2] is the stability index, β∈[−1,1] is the skewness, σ∈(0,∞) is the scale parameter and μ∈R is the shift parameter. For α=2 it reduces to the Gaussian distribution with the variance equal to $2\sigma^{2}$ and the mean μ. In this case, the skewness parameter β does not affect the result. It is important to note that σ is not equal to the standard deviation. If the β=0 and μ=0 this distribution simplifies to the symmetric α-stable (denoted SαS), which is the form used in the proposed model. In the α-stable distribution, the α parameter is the most important. When the α decreases, the tails are going to be heavier. This can be observed in the behavior of the signal as the appearance of more frequent impulses with higher values.

According to the scheme presented in Figure 7, the following components are used to compose the signal:

- the main sine component is related to the rotation of the shaft,
- the Gaussian noise is related to general external environmental conditions,
- the transfer function H(t) (see Equation (5)) is prepared to obtain the response of the machine to the falling ore pieces,
- the convolution of the Gaussian noise and the transfer function H(t) is performed to obtain the response of the machine to the external noise,
- the convolution of the α-stable noise (which imitates the large observations in the signal related to falling oversized lumps) with the transfer function H(t) is performed to obtain the response of the machine to the high-energy impact excitations.

Additionally, noise components, both processed and not processed by the machine transfer function, are subjected to amplitude modulation, where the modulating function is a sine wave.

## 4. Results

In this section, the results of the intermediate steps of the described procedure are presented.

**Figure 8:**
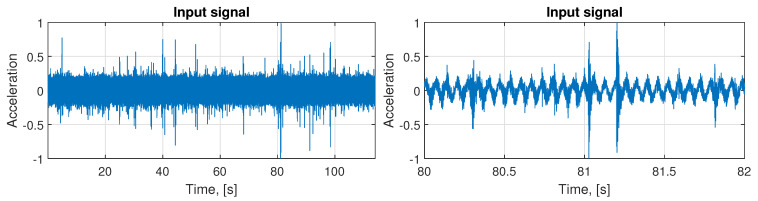
The raw input signal (left panel) and its two seconds part (right panel).

The exemplary normalized real signal comes from the spring (located in the right lower part, see Figure 4) measured on the vibration screen with the sampling frequency $f_{s}=$ 25,000 Hz. It contains 2,850,000 samples, which translates to 114 s. In Figure 8 on the left panel, we can observe the analyzed signal, and on the right panel, its part between 80th to 82nd second.

**Figure 9:**
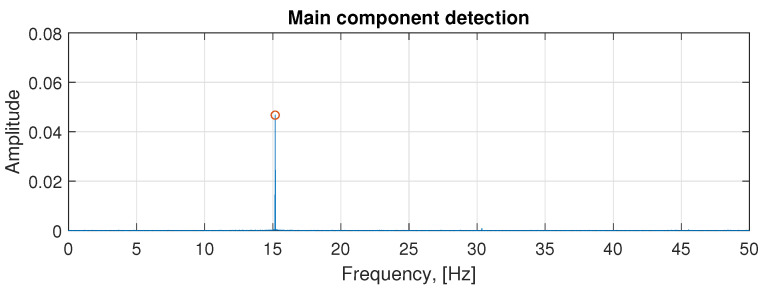
Main sine component detected in the signal clearly visible on the Fourier spectrum.

First, in Figure 9, the amplitude spectrum of the real input signal is presented. Although the spectrum appears to be extremely clean, we would like to assure the reader that the scale of the plot is suppressing the entire content present along the spectrum, and only the main sine component is visible due to its overwhelming amplitude value compared to the rest of frequency components.

**Figure 10:**
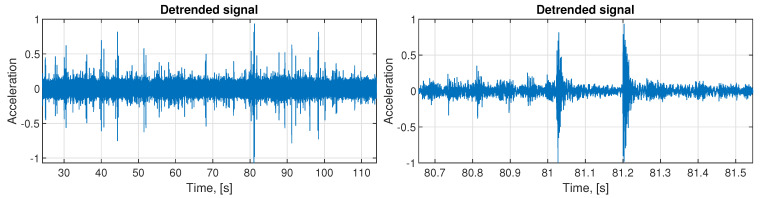
The signal after the main sine subtraction (**left**) and its short part for better visibility (**right**).

**Figure 11:**
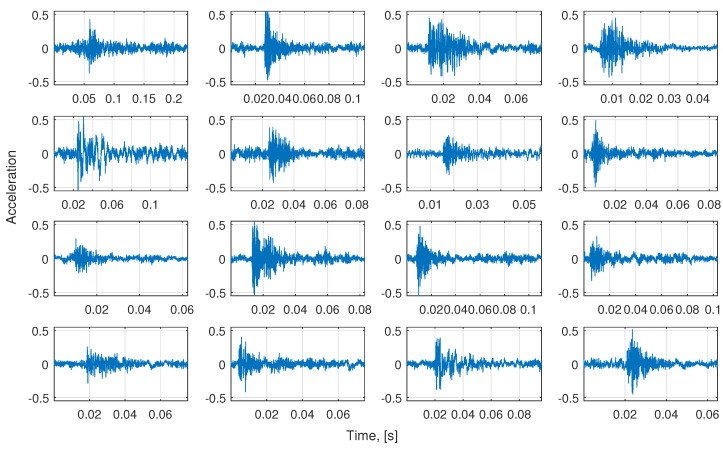
The exemplary impulses selected from the de-trended signal.

In Figure 10 the whole de-trended signal is presented (left panel) and its zoom (right panel). It was obtained by subtracting the main sine component from the input data presented in Figure 8. Based on this signal, we select 68 segments with representative examples of impulses. The exemplary set of these impulses is presented in Figure 11. As one can see, the impulses are not identical, but there is no clear way to differentiate them basing on the visual evaluation. However, there are present impulses that belong to both classes, which are different from each other. This is why it is required to group them using the clustering approach, even if the differences are not obvious.

**Figure 12:**
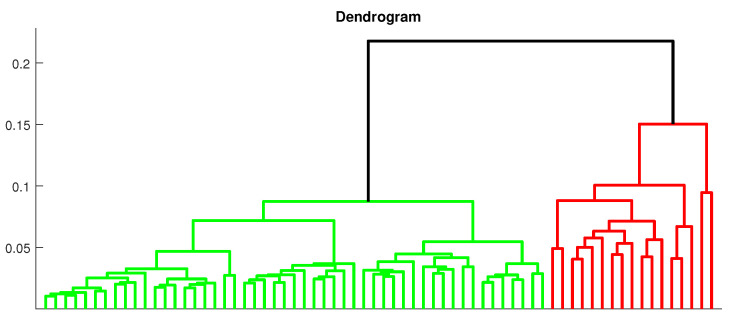
The dendrogram for the spectra corresponding to the 68 segments presents the results of the hierarchical clustering.

In Figure 12 the dendrogram is presented. It is a way to visually present the structure of clustered data produced by hierarchical clustering. It allows us to confirm the result obtained in the Silhouette criterion that two classes are optimal. They are marked in the dendrogram in green and red color. One can see that even visual evaluation of the distance between two main clusters confirms the division, and that the density of subclusters packing suggests high intracluster similarity. After we use the hierarchical clustering for the spectra of these 68 segments, we obtain classes with 51 and 17 vectors.

**Figure 13:**
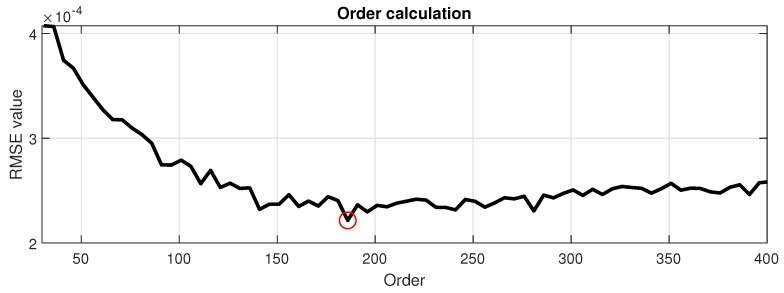
The values of RMSE error for predefined range of AR order *p*.

For the order calculation, we use the most numerous class (51 vectors) obtained by the clustering. The value of RMSE for each of the analyzed order can be observed in Figure 13. The order of the AR model is selected based on the minimal value of RMSE in the analyzed range, which turned out to be p=185 (marked with a red circle on the plot). It is interesting to notice that, above this value, the trend of errors starts to ascend, and the selected value lies in a global minimum.

**Figure 14:**
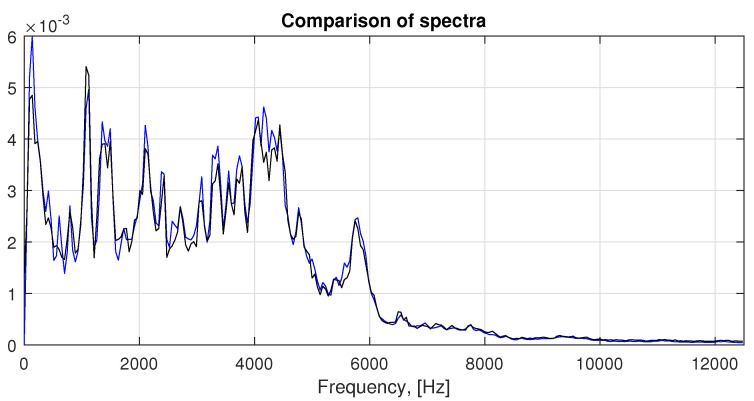
Comparison of spectra for both classes. Blue line: spectrum of signal obtained from averaged autoregressive (AR) coefficients. Black line: averaged spectrum of segments from real signal.

In Figure 14 the comparison of spectra is presented in accordance to the description in Section 3.3. The averaged spectrum of segments from the real signal is marked by black color and the spectrum obtained from the modeled signal is marked by blue color. The spectrum obtained from the model describes the general behavior relatively well.

**Figure 15:**
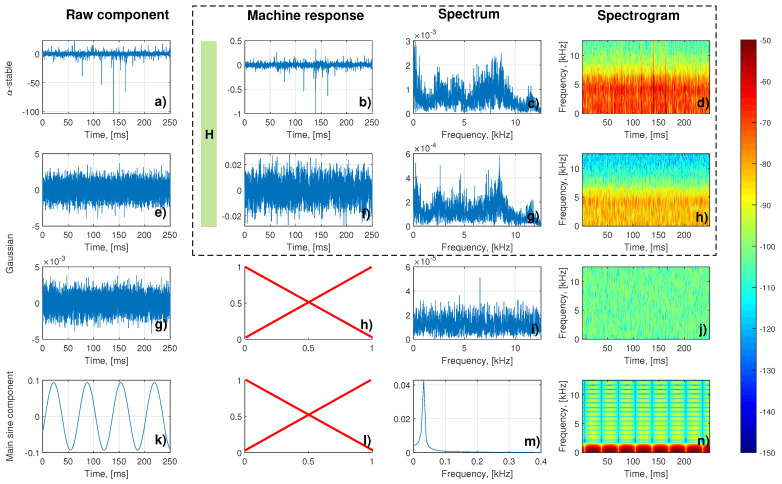
The components of the model mentioned in the scheme (Figure 7).

Finally, it was possible to construct the signal from the described components (see Figure 15) according to the scheme presented in Figure 7. Four columns of this chart represent respectively:

- Raw component presented as time series: ore excitation (Figure 15a), internal Gaussian noise (Figure 15e), external Gaussian noise (Figure 15g), main sine component (Figure 15k);
- Target component (raw components processed by machine transfer function if applicable (Figure 15b,f); external noise (Figure 15g) and sine component (Figure 15k) are not processed);
- Fourier spectrum of the target components (Figure 15c,g) and raw components (Figure 15i,m);
- Spectrograms of the target components (Figure 15d,h) and raw components (Figure 15j,n)

**Figure 16:**
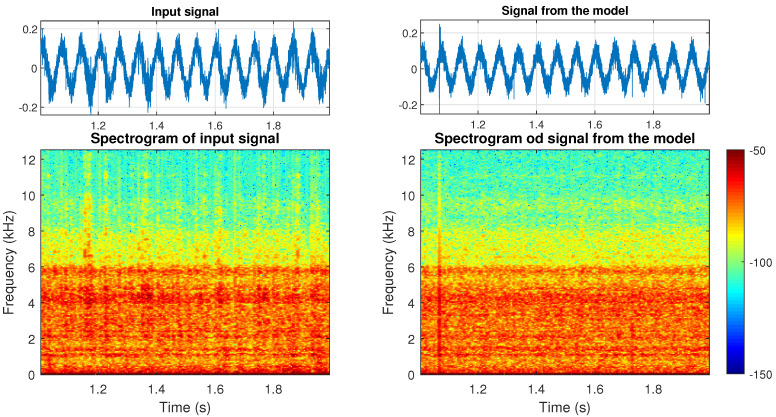
The one second of the time series and spectrogram of input signal (**left**) and signal from the model (**right**).

Based on this figure one can see how important it is to use machine response as a part of the overall model. Firstly, Gaussian noise used as excitation for machine response (which simulates internal noises) resembles the background of the real signal very closely, which is the expected outcome (Figure 15e–h). It is especially visible comparing spectrograms of the simulated internal noises (Figure 15h) and the actual real signal (Figure 16 bottom-left panel). Similarly, process drawn from α-stable distribution (Figure 15a) used as excitation for machine response (which simulates ore-induced noises, see spectrogram at Figure 15d) takes a similar spectral shape, but is focused on describing the elementary excitations as time passes, which explains its irregular structure in the time domain.

In Figure 16 on the left side, the time series and spectrogram for a one second of the real signal are presented, and on the right side—time series and spectrogram of the signal generated based on the presented model. The small differences between spectrograms can be observed. They are connected with shocks observed in the real data. In our model, the size of shocks depends on the α∈(0,2] parameter from the α-stable distribution. When α is close to 2, the shocks do not exist. As α decreases, the impulses becomes larger. The model presented in Figure 16 corresponds with α=1.94 (see the first row in Figure 15), because such value corresponds the best with the features of the real signal taken into consideration for constructing this particular realization of the model. However, one has to remember that values can differ when a different specific signal is taken into modeling procedure, which is understandable, e.g., the value of α can be closer to 2 if in given signal segment there are not many large ore pieces, and signal will not be as impulsive.

## 5. Conclusions

In this paper, the initial approach to constructing the signal model of the industrial vibrating sieving screen suspension vibrations has been presented. The model is constructed based on the analysis and decomposition of the real signal measured on the actual machine operating in the mining industry.

Firstly, the main harmonic behavior of the machine shaft rotation is described with a single sine wave. Next, the simulation of the expected mechanical structure excitation (impacts) by ore stream lumps is described by using the α-stable process. The autoregressive model matched single impulse in order to describe impulse response of the machine. Finally, internal and external noises are included, represented by Gaussian noise processed and unprocessed by the AR model, used to properly describe the spectral structure of signal background.

There are many models of vibration signals from machines in the literature. However, modeling the vibration signal from the sieving screen is a special case. The falling copper ore lumps cause a highly impulsive contribution to the signal. In the presented model, the α-stable distribution is processed by the transfer function to obtain the response of the machine to the high-energy impact excitations. Furthermore, by changing the α parameter, the behavior of obtained noise can be controlled.

The method of constructing the model of the signal based on the vibration signal proposed in this paper has also some limitations. They can be classified into two groups. Firstly, the main sinusoidal component related to the rotation of the shaft should be detected during the first step. However, the number and size of falling pieces of the ore are variable over time and can affect the operational parameters of the machine, causing in this way a change in the parameters of the main deterministic component—the sine. It can make its parameters time variable and require more advanced methods to estimate the main component in the proper way. Fortunately, in the presented case this problem does not exist (the material stream was relatively stationary here). Secondly, since the AR model is estimated for selected and representative impulses, the proposed procedure cannot be used if the excitations are not observed in the signal. However, for such cases, there are many models described extensively in other works. Additionally, it is possible that the measured signal has impulsive behavior but it is hard to find the segments of representative impulses (e.g., some of the impulses can overlap and cannot be taken as a representative one).

This work is a first step towards understanding of the motion and vibrations characteristic to this type of machines. This, and similar models, i.e., for signals recorded on the bearings of a machine of this type, can allow to introduce model-based diagnostics of the various screen elements. In further work, authors intend to take into consideration additional time-varying parameters, such as uneven flow of material, uneven total load etc., since for now the model assumes stationary conditions. Authors also intend to validate the model using a larger amount of real-life data, measured on the rest of the springs as well as the one used in this work. Additionally, similar models will be described for signals measured on other parts of the machine. The modeling procedure will be in this case analogous, however all the parameters need to be tuned individually.

It is important to note that the model presented in this paper simulates the signal without any faults, because the machine is in the healthy condition. It is believed, that such model can be used for condition monitoring purposes. When the signal will contain fault related component, it will not be included in the model, so final residual will have specific to local fault behaviour. However, until now this has not been validated yet, so we plan this as near future research. 

## Data Availability

Data are not available due to non-disclosure agreements.
